# Visualization of endogenous G proteins on endosomes and other organelles

**DOI:** 10.1101/2024.03.05.583500

**Published:** 2024-03-07

**Authors:** Wonjo Jang, Kanishka Senarath, Sumin Lu, Nevin A. Lambert

**Affiliations:** Department of Pharmacology and Toxicology, Medical College of Georgia, Augusta University, Augusta, GA, 30912, USA

## Abstract

Classical G protein-coupled receptor (GPCR) signaling takes place in response to extracellular stimuli and involves receptors and heterotrimeric G proteins located at the plasma membrane. It has recently been established that GPCR signaling can also take place from intracellular membrane compartments, including endosomes that contain internalized receptors and ligands. While the mechanisms of GPCR endocytosis are well understood, it is not clear how internalized receptors are supplied with G proteins. To address this gap we use gene editing, confocal microscopy, and bioluminescence resonance energy transfer to study the distribution and trafficking of endogenous G proteins. We show here that constitutive endocytosis is sufficient to supply newly internalized endocytic vesicles with 20-30% of the G protein density found at the plasma membrane. We find that G proteins are present on early, late, and recycling endosomes, are abundant on lysosomes, but are virtually undetectable on the endoplasmic reticulum, mitochondria, and the medial Golgi apparatus. Receptor activation does not change heterotrimer abundance on endosomes. Our results provide a detailed subcellular map of endogenous G protein distribution, suggest that G proteins may be partially excluded from nascent endocytic vesicles, and are likely to have implications for GPCR signaling from endosomes and other intracellular compartments.

## Introduction

Heterotrimeric G proteins transduce a vast number of important physiological signals (Gilman, 1987), most often in response to activation by G protein-coupled receptors (GPCRs) (Pierce, Premont, & Lefkowitz, 2002). Canonical G protein signaling occurs when a cell surface GPCR is activated by an extracellular ligand, which in turn promotes activation of plasma membrane G protein heterotrimers and downstream effectors. Recently, it has become clear that GPCRs can also signal from intracellular compartments (Calebiro et al., 2010; Eichel & von Zastrow, 2018), most notably endosomes and the Golgi apparatus (Calebiro et al., 2009; Ferrandon et al., 2009; Irannejad et al., 2017; Irannejad et al., 2013; Mullershausen et al., 2009). Signaling from endosomes is often a continuation of signaling that starts at the plasma membrane and persists as (or resumes after) active receptors are endocytosed (Tsvetanova, Irannejad, & von Zastrow, 2015). Much is known about the machinery responsible for GPCR internalization, and also about the trafficking itineraries of specific receptors after endocytosis. Some receptors are efficiently sorted for recycling and are returned to the plasma membrane, whereas other receptors are rapidly degraded (Hanyaloglu & von Zastrow, 2008). It is also known that at least one isoform of the G protein effector adenylyl cyclase is actively internalized (Lazar et al., 2020).

In contrast, much less is known about how G protein heterotrimers traffic from the plasma membrane through intracellular compartments (Wedegaertner, 2012). It is not known how efficiently heterotrimers are loaded onto endocytic vesicles at the plasma membrane, how receptor activation might change this process, or what the fate of G proteins might be after endocytosis. Activation at the plasma membrane promotes heterotrimer dissociation, and the resulting loss of membrane avidity allows Gβγ dimers and some Gα subunits to translocate through the cytosol to sample intracellular membranes (Akgoz, Kalyanaraman, & Gautam, 2004; Hynes et al., 2004; Ransnäs et al., 1989; Slepak & Hurley, 2008; Wedegaertner, Bourne, & von Zastrow, 1996). However, these processes reverse quickly when activation ceases (Akgoz, Kalyanaraman, & Gautam, 2004), meaning that activation-dependent translocation of free Gα subunits and Gβγ dimers would be an inefficient mechanism to deliver inactive heterotrimers to intracellular membranes. While G proteins have been detected on the surface of endosomes and other intracellular compartments (Hewavitharana & Wedegaertner, 2012; Irannejad et al., 2013; Scarselli & Donaldson, 2009; Wedegaertner, 2012) there has been no quantitative comparison of G protein distribution across subcellular compartments.

Here we study the subcellular distribution of endogenous heterotrimeric G proteins in cultured cells using CRISPR-mediated gene editing, confocal imaging, and bioluminescence resonance energy transfer (BRET). We find that G proteins are abundant on membrane compartments that are functionally continuous with the plasma membrane, including early, late, and recycling endosomes. However, heterotrimer density on endocytic membranes is lower than on the plasma membrane, suggesting that G protein endocytosis is inefficient. Endocytic trafficking of G proteins is not regulated by GPCRs. Our findings are likely to have implications for GPCR signaling from endosomes, as internalized receptors are concentrated in G protein-deficient compartments.

## Results

**Figure 1:**
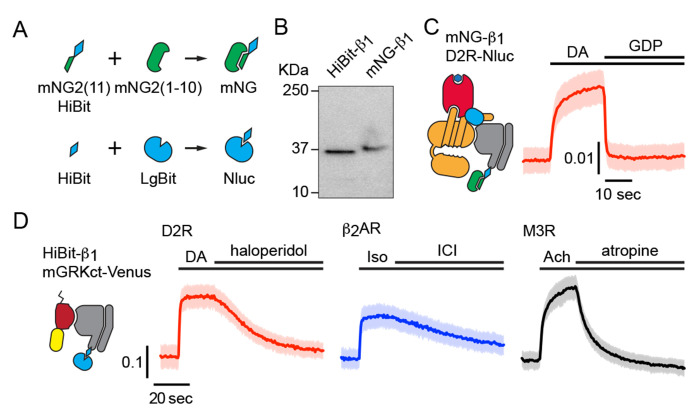
Validation of mNG-β_1_ and HiBit-β_1_ cells. (**A**) Cartoon showing the peptide tag complementation systems used to label endogenous Gβ_1_ subunits. (**B**) SDS-PAGE of HiBit-β_1_ and mNG-β_1_ cell lysates; the predicted molecular weights of the edited gene products are 38.9 and 41.1 kilodaltons (KDa), respectively; representative of 3 independent experiments. (**C**) In permeabilized nucleotide-depleted cells BRET between dopamine D2R-Nluc receptors and mNG-β_1_-containing heterotrimers increases in response to dopamine (DA; 100 μM) and reverses after addition of GDP (100 μM); mean ± 95% CI; *n*=27 replicates from 2 independent experiments. (**D**) In intact cells BRET between HiBit-β_1_ and the Gβγ sensor memGRKct-Venus increases after stimulation of D2R dopamine, β_2_AR adrenergic, or M3R acetylcholine receptors with DA (100 μM), isoproterenol (Iso; 10 μM) and acetylcholine (Ach; 100 μM), respectively. Signals reversed when receptors were blocked with haloperidol (10 μM), ICI 118551 (10 μM) or atropine (10 μM); mean ± 95% CI; *n*=16 replicates from 4 independent experiments.

To study the localization of endogenous G proteins we used gene editing to attach small peptide tags to the amino terminus of Gβ_1_ subunits (*GNB1*) in HEK 293 cells. We chose this subunit because it is the most abundant Gβ subunit in this cell type (Cho et al., 2022), it can associate with any type of Gα or Gγ subunit (Hillenbrand et al., 2015), and it can be labeled without disrupting heterotrimer formation or function. For bioluminescence experiments we added the HiBit tag (Schwinn et al., 2018) and isolated clonal “HiBit-α_1_” cell lines. For imaging experiments we added a tandem tag that included the 11^th^ beta strand of mNeonGreen2 (mNG2(11)) (Feng et al., 2017) and HiBit in cells constitutively expressing mNG2(1-10) and isolated “mNG-α_1_” cell lines (Figure 1A). Amplicon sequencing verified that cell lines had correctly edited *GNB1* genes and SDS-PAGE revealed single proteins with apparent molecular weights consistent with edited Gβ_1_ subunits (Figure 1B). BRET assays demonstrated that tagged subunits in HiBit-β_1_ and mNG-β_1_ cell lines formed functional heterotrimers with endogenous Gα and Gγ subunits (Figure 1C, D). Endogenous Gα and Gβ subunits are expressed at approximately a 1:1 ratio, and Gβ subunits are tightly associated with Gγ and inactive Gα subunits (Cho et al., 2022; Gilman, 1987; Krumins & Gilman, 2006), therefore we assume that the large majority of mNG-β_1_ and HiBit-β_1_ subunits in unstimulated cells are part of heterotrimers.

### Endogenous G proteins primarily associate with the plasma membrane and endolysosomes

**Figure 2:**
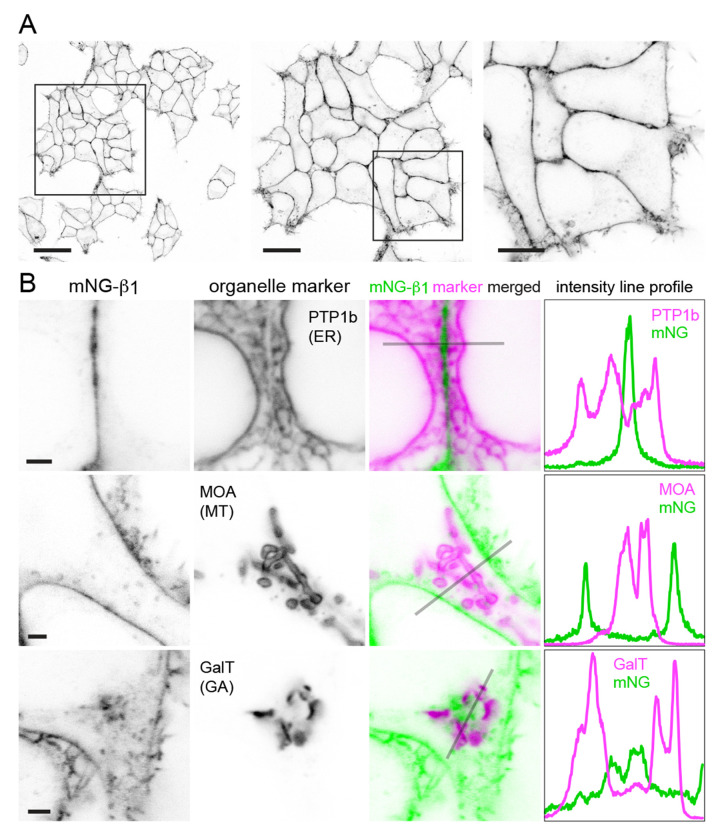
Endogenous G proteins are abundant on the plasma membrane but not large organelles. (**A**) A single field of view of mNG-β_1_ cells at three magnifications; scale bars are 40 μm, 20 μm and 10 μm. (**B**) mNG-β_1_ does not colocalize with expressed markers of the endoplasmic reticulum (ER; PTP1b), mitochondria (MT; MOA) or medial Golgi apparatus (GA; GalT); intensity line profiles depict absolute fluorescence intensity in each channel; scale bars are 2 μm.

Confocal imaging of mNG-β_1_ cells revealed the expected bright fluorescence at the plasma membrane. Most cells also contained pleiomorphic intracellular structures and dim cytosolic fluorescence that was sufficient to suggest relative exclusion of mNG-β_1_ from the nucleus. Especially notable were clusters of large vesicular structures located at the cell periphery which were later identified as lysosomes (Figure 2A; see below). Large intracellular organelles such as the endoplasmic reticulum, mitochondria, and Golgi apparatus were not evident.

To identify the intracellular membrane compartments with mNG-β_1_ fluorescence we coexpressed a series of organelle markers tagged with red fluorescent proteins. Markers of the endoplasmic reticulum, mitochondria and medial Golgi apparatus indicated that these large compartments were virtually devoid of mNG-β_1_ fluorescence (Figure 2B–D, Figure 2 - figure supplements 1–3). In some cells an indistinct region of mNG-β_1_ fluorescence was interleaved with leaflets of the Golgi apparatus, but line profiles suggested that this was a distinct structure (Figure 2D, Figure 2 - figure supplement 3), most likely the perinuclear recycling compartment (see below).

**Figure 3:**
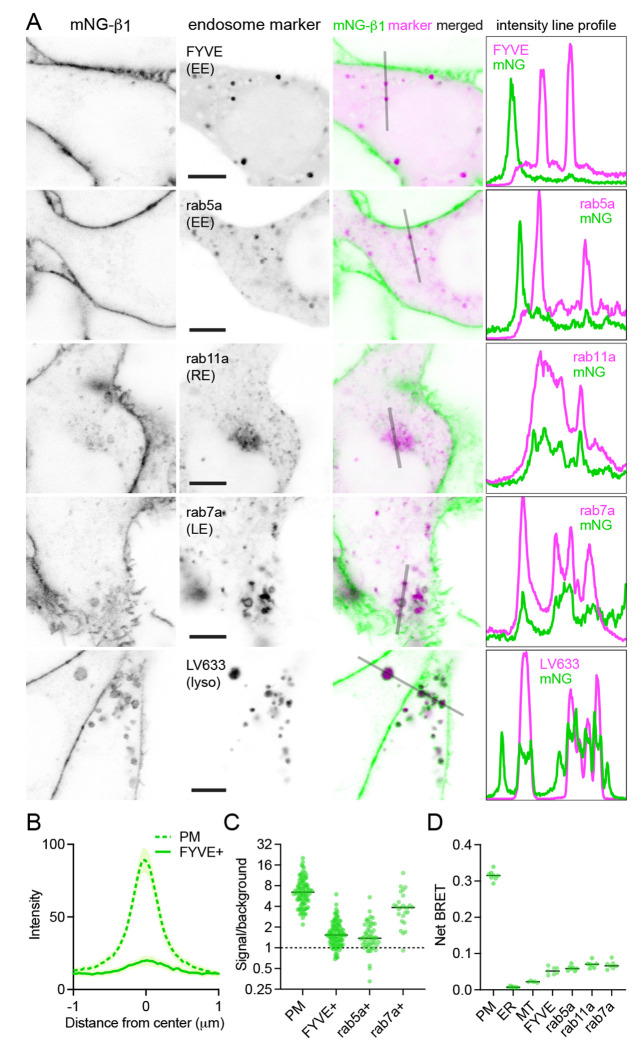
Endogenous G proteins colocalize with markers of endosomes and lysosomes. (**A**) mNG-β_1_ colocalizes with expressed markers of early endosomes (EE; FYVE and rab5a), recycling endosomes (RE; rab11a), late endosomes (LE; rab7a) and lysosomes (lyso; LysoView 633); intensity line profiles depict absolute fluorescence intensity in each channel; scale bars are 5 μm. (**B**) Mean mNG-β_1_ fluorescence intensity line profiles drawn across the plasma membrane (PM) and FYVE-positive vesicles; mean ± 95% CI; *n*=40 vesicles/cells. (**C**) mNG-β_1_ signal/background ratios for regions of interest surrounding the plasma membrane (PM; *n*=99), FYVE-positive (*n*=125) and rab5a-positive (*n*=56) early endosomes, and rab7a-positive (*n*=26) late endosomes; horizontal lines represent the median. (**D**) Bystander net BRET signals between HiBit-β_1_ and Venus-tagged markers of the plasma membrane (PM), endoplasmic reticulum (ER), mitochondria (MT), early endosomes (FYVE and rab5a), recycling endosomes (rab11a) and late endosomes (rab7a); horizontal lines represent the median; *n*=5-7 independent experiments.

In contrast, mNG-β_1_ clearly colocalized with the marker FYVE, which binds to phosphatidylinositol-3-phosphate (PI3P) on the surface of endosomes (Figure 3A, B, Figure 3 - figure supplement 1). However, mNG-β_1_ fluorescence was not detected on every FYVE-positive vesicle and when present was much less intense than fluorescence of adjacent segments of the plasma membrane (Figure 3B). The median signal-to-background ratio for FYVE-positive structures was less than one-fourth that of the plasma membrane (Figure 3C). FYVE domains primarily localize to early endosomes (Hammond & Balla, 2015), so it was not surprising that similar colocalization of mNG-β_1_ was observed with the early endosome marker rab5a. As was the case with FYVE, mNG-β_1_ was detectable in some rab5a-positive vesicles but not others and was not as intense as the nearby plasma membrane (Figure 3A, C, Figure 3 - figure supplement 2). In order to determine the fate of G proteins after endocytosis we then examined mNG-β_1_ colocalization with markers of recycling and late endosomes (Stenmark, 2009). Dim mNG-β_1_ fluorescence was detected on indistinct rab11a-positive structures clustered diffusely in the vicinity of the nucleus (Figure 3A, Figure 3 - figure supplement 3), which we presumptively identified as the perinuclear recycling compartment (PNRC). Similarly, mNG-β_1_ colocalized extensively with vesicles labeled with rab7a, a marker of late endosomes (Figure 3A, Figure 3 - figure supplement 4). Notably, mNG-β_1_ fluorescence was more intense on rab7a-positive late endosomes than on FYVE- or rab5a-positive early endosomes (Figure 3C). The presence of mNG-β_1_ on late endosomes suggested that some G proteins may be degraded by lysosomes. Accordingly, mNG-β_1_ strongly colocalized with lysosomes marked with LysoView 633 (Figure 3A, Figure 3 - figure supplement 5) or long-term incubation with fluorescent dextran (Figure 3 - figure supplement 5). In many instances the intensity of mNG-β_1_ fluorescence on lysosomes was similar to that of the nearby plasma membrane (Figure 3A). These imaging results suggest that G proteins are likely to undergo endocytosis and enter both recycling and degradative pathways and may become more concentrated as late endosomes mature.

As an alternative approach we performed bystander BRET experiments to map the subcellular localization of endogenous HiBit-β_1_. This approach provides an unbiased index of membrane protein colocalization from large populations of cells and has the additional advantage of very high sensitivity (Lan et al., 2012). We expressed LgBit and a series of inert Venus-tagged membrane markers in HiBit-β_1_ cells and observed large bystander signals at the plasma membrane, smaller bystander signals at endosomes, and very small bystander signals at the endoplasmic reticulum and mitochondria (Figure 3D). Although bystander BRET signals cannot be directly compared between different compartments, these results are generally consistent with what we observed using confocal imaging and confirm the presence of G proteins on multiple endosomal compartments.

### Constitutive G protein endocytosis is inefficient

**Figure 4:**
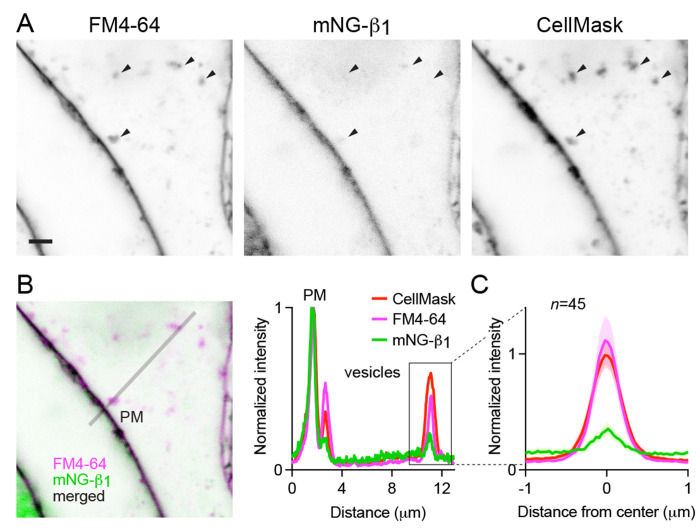
Constitutive G protein endocytosis is inefficient. (**A**) mNG-β_1_ colocalizes with newly internalized endocytic vesicles labeled with FM4-64 and CellMask Deep Red (arrowheads); scale bar is 2 μm. (**B**) A fluorescence intensity line profile for mNG-β_1_, FM4-64 and CellMask normalized to the peak value of each label at the plasma membrane (PM). (**C**) Mean mNG-β_1_, FM4-64 and CellMask fluorescence intensity line profiles drawn across vesicles, normalized to fluorescence intensity at the plasma membrane for each label; mean ± 95% CI; *n*=45 vesicles/cells.

That mNG-β_1_ fluorescence was less intense on endosomes than the plasma membrane suggested that G protein density may be lower on the surface of endosomes than on the plasma membrane. However, differences in fluorescence intensity could be due to differences in the amount of membrane surface area sampled in the imaging volume. Likewise, differences in bystander BRET between compartments could be due to differences in several factors, including expression and efficiency of compartment-specific BRET acceptors. Therefore, we devised a co-labeling protocol that allowed us to compare mNG-β_1_ fluorescence to the amount of newly internalized membrane imaged at endocytic vesicles, and to make the same measurements at the plasma membrane. To stain both the plasma membrane as well as newly formed endocytic vesicle membrane we exposed live cells to the styryl dye FM4-64, which rapidly and reversibly partitions into (but does not cross) membranes and is only fluorescent in a hydrophobic environment (Betz, Mao, & Smith, 1996). When cells are exposed to FM4-64 at physiological temperatures the plasma membrane is stained immediately, and this is followed over the course of several minutes by the appearance of intracellular vesicles that have trapped the dye (Figure 4A, Figure 4 - figure supplement 1). As an orthogonal approach we stained cells with CellMask Deep Red, a lipophilic dye that permanently stains the plasma membrane and therefore is incorporated into endocytic vesicles. Both dyes are expected to produce fluorescence signals proportional to the surface area of the membrane sampled by the imaging method, allowing us to normalize the fluorescence of individual vesicles to the nearby plasma membrane. We reasoned that if G proteins are passively incorporated into endocytic vesicles without any enrichment or exclusion, then mNG-β_1_ fluorescence in each vesicle should have the same intensity relative to the plasma membrane as lipophilic dyes. After staining cells and allowing 15 minutes for constitutive endocytosis we found that FM4-64 and CellMask dyes reported similar amounts of membrane surface area in endocytic vesicles (Figure 4A, B); in both cases peak vesicle intensity was on average similar to the intensity of the plasma membrane (Figure 4C). In contrast, peak mNG-β_1_ fluorescence on the same endocytic vesicles was much less intense than the plasma membrane (Figure 4A-C). There was considerable variability between individual vesicles, such that some vesicles contained no detectable mNG-β_1_ fluorescence (Figure 4A, Figure 4 - figure supplement 1). This result confirms that heterotrimeric G proteins are present on newly internalized membrane but also suggests that G proteins are partially excluded from endocytic vesicles.

### Receptor activation does not change G protein endocytosis

**Figure 5:**
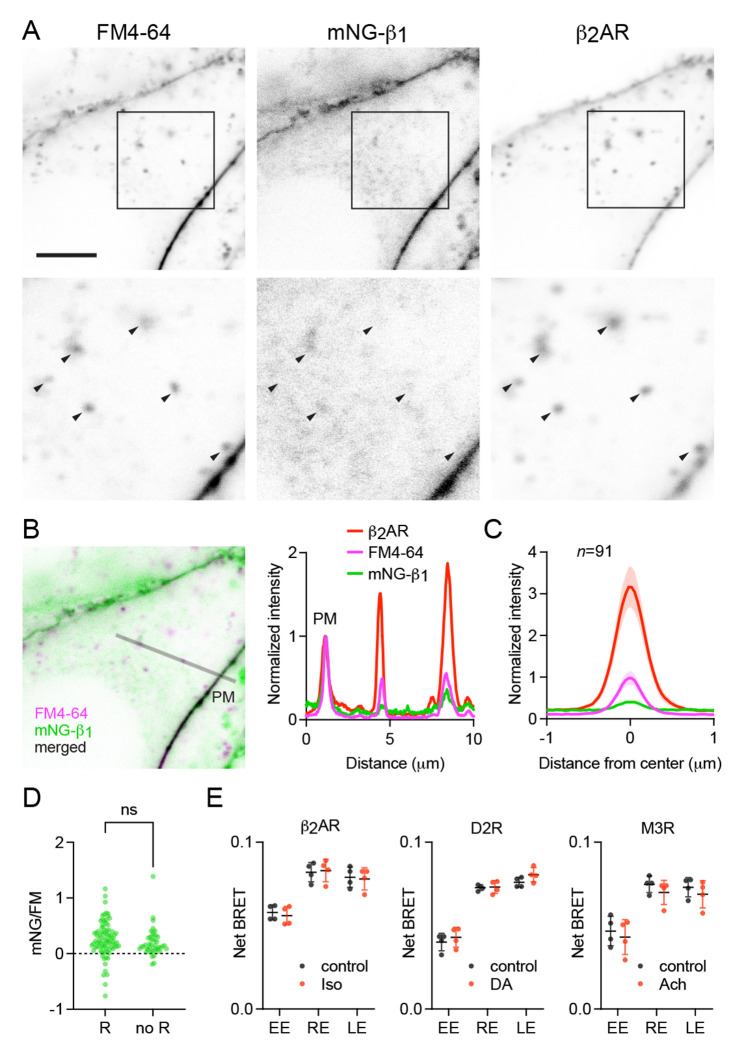
Receptor activation does not change G protein endocytosis. (**A**) mNG-β_1_ colocalizes with newly internalized endocytic vesicles labeled with FM4-64 and SNAP-tagged β_2_ adrenergic receptor (β_2_AR) labeled with Alexa Fluor 674; scale bar is 5 μm. Cells were stimulated with 10 μM isoproterenol for 15 minutes to induce β_2_AR internalization. (**B**) A fluorescence intensity line profile for mNG-β_1_, FM4-64 and β_2_AR normalized to the peak value of each label at the plasma membrane (PM). (**C**) Mean mNG-β_1_, FM4-64 and β_2_AR fluorescence intensity line profiles drawn across vesicles, normalized to fluorescence intensity at the plasma membrane for each marker; mean ± 95% CI; *n*=91 vesicles/cells. (**D**) Peak mNG-β_1_ signals divided by peak FM4-64 signals (each normalized to the plasma membrane) did not differ between vesicles that contained receptors (R; *n*=91) and vesicles formed by constitutive endocytosis (no R; *n*=45); n.s., not significant, P=0.20, unpaired t-test. (**E**) Bystander BRET between HiBit-β_1_ and Venus-tagged markers of early endosomes (EE; rab5a), recycling endosomes (RE; rab11a) and late endosomes (LE; rab7a) was unchanged after 30 minutes of receptor activation with isoproterenol (Iso; 10 μM), dopamine (DA; 100 μM) or acetylcholine (Ach; 100 μM); mean ± SD, *n*=4 independent experiments; no agonist-treated group was significantly different from the control, paired t-test with a false discovery rate (FDR) of 1%.

The above results suggested that constitutive endocytosis of heterotrimeric G proteins is inefficient. However, it is possible that GPCR and G protein activation could change how G proteins are loaded onto endocytic vesicles. To test this possibility, we performed similar imaging experiments with mNG-β_1_ cells transfected with SNAP-tagged β_2_ adrenergic receptors (SNAPf-β_2_AR). This receptor is often used as a model of activity-dependent GPCR internalization (Benovic et al., 1988; von Zastrow & Kobilka, 1992) and has been shown to activate G proteins on endosomes (Bowman, Shiwarski, & Puthenveedu, 2016; Irannejad et al., 2013). We labeled SNAPf-β_2_AR with a membrane-impermeant SNAP ligand (AF 647) at room temperature to prevent constitutive endocytosis, then incubated cells with FM4-64 and the agonist isoproterenol for 15 minutes at physiological temperature to promote receptor endocytosis. Confocal imaging after agonist washout revealed numerous intracellular vesicles with intense AF 647 fluorescence, consistent with robust receptor internalization (Figure 5A). Normalization and comparison to FM4-64 fluorescence indicated that SNAPf-β_2_AR was enriched approximately three-fold on endocytic vesicles compared to the nearby plasma membrane (Figure 5B, C), consistent with active recruitment of active receptors to clathrin-coated pits and endocytic vesicles. In contrast, mNG-β_1_ fluorescence in the same vesicles was again lower than expected given the amount of membrane imaged in each vesicle (Figure 5B, C). Once again there was considerable variability between individual endocytic vesicles (Figure 5A, D). Using FM4-64 fluorescence as a standard for membrane surface area we calculated that mNG-β_1_ density on receptor-containing vesicles was 28 ± 8% (mean ± 95% CI; *n*=91) of the nearby plasma membrane. Although this density was higher than that calculated for vesicles formed by constitutive endocytosis (20 ± 8%; *n*=45), the difference did reach significance (Figure 5D). These results demonstrate that activation-dependent internalization of β_2_ adrenergic receptors does not significantly promote or prevent loading of G proteins onto endocytic vesicles.

These findings suggested that receptor activation should have no impact on the abundance of G proteins on endosomes. To test this idea, we performed bystander BRET experiments with HiBit-β_1_ cells transiently expressing β_2_ adrenergic, D2 dopamine or M3 muscarinic receptors to activate G_s_, G_i/o_ and G_q/11_ heterotrimers, respectively. We incubated cells with agonist for 30 minutes under conditions permissive for vesicular trafficking, then washed with antagonist to allow receptors and heterotrimers to become inactive prior to measuring BRET. Under these conditions no significant changes in bystander BRET were observed at any endosome compartment (Figure 5E). These results support the idea that receptor and G protein activation do not lead to persistent changes in G protein abundance on the surface of endosomes.

## Discussion

While the mechanisms involved in the biosynthesis, chaperoning and trafficking of nascent G protein heterotrimers are fairly well understood (Gabay et al., 2011; Marrari et al., 2007; Wedegaertner, 2012), the mechanisms that regulate the subcellular distribution of heterotrimers after delivery to the plasma membrane have not been studied as extensively. Here we show that constitutive and activity-dependent endocytosis of G proteins is inefficient. Avoidance of endocytosis is likely to be important for maintaining a high density of heterotrimers at the plasma membrane, where much important signaling takes place. On the other hand, this limits the abundance of G proteins on the surface of endosomes. At present we can only speculate regarding the mechanism that limits G protein density on endocytic vesicles. Many endocytosis mechanisms, including clathrin-mediated endocytosis, rely on bulky coat proteins and adapters to induce membrane curvature and recruit cargo (Doherty & McMahon, 2009). One possibility is that heterotrimeric G proteins are simply excluded from nascent endocytic vesicles by steric occlusion. While large extracellular domains are known to impede endocytosis of membrane proteins (DeGroot et al., 2018), a similar relationship has not been demonstrated for intracellular domains. It is noteworthy that the monomeric G proteins H-Ras and N-Ras are also less abundant on endosomes than the plasma membrane, and therefore are separated from internalized growth factor receptors (Pinilla-Macua, Watkins, & Sorkin, 2016; Surve, Watkins, & Sorkin, 2021).

Some studies using overexpressed G protein subunits have suggested that a large pool of G proteins is located on intracellular membranes, including the Golgi apparatus (Chisari et al., 2007; Saini et al., 2007; Tsutsumi et al., 2009), whereas others have indicated a distribution that is dominated by the plasma membrane (Crouthamel et al., 2008; Evanko, Thiyagarajan, & Wedegaertner, 2000; Marrari et al., 2007; Takida & Wedegaertner, 2003). A likely factor contributing to this discrepancy is the stoichiometry of overexpressed subunits, as neither Gα nor Gβγ traffic appropriately to the plasma membrane as free subunits (Wedegaertner, 2012). Our results show that endogenous G proteins are primarily located on the plasma membrane and are present on internal membranes at substantially lower levels. We identify the specific intracellular compartments where G proteins are found and show the relative abundance of G proteins on each compartment. Nascent heterotrimers are likely formed and lipid modified on the endoplasmic reticulum and Golgi apparatus (Wedegaertner, 2012), yet few heterotrimers can be found on these compartments at any given moment, consistent with a relatively slow rate of turnover compared to forward trafficking during biosynthesis (Gabay et al., 2011). In the present study we have limited our analysis to the medial portion of the Golgi apparatus. It is possible that G proteins may be more abundant on the trans-Golgi network, as this compartment is involved in membrane protein recycling (Nakano, 2022). Likewise, we found that few heterotrimers are associated with mitochondria, despite the fact that previous studies have demonstrated functional roles for G proteins on these organelles (Hewavitharana & Wedegaertner, 2012). Our results suggest that GPCR signaling from intracellular compartments will generally have to be transduced by a lower density of G proteins.

Fully lipid-modified heterotrimers in their inactive state are unlikely to detach from membranes at a significant rate (Shahinian & Silvius, 1995). Therefore, we infer from the presence of Gβ_1_ on early, late, and recycling endosomes that heterotrimers undergo vesicle-mediated endocytosis in unstimulated cells and are not efficiently sorted to either the slow recycling pathway or the degradative pathway. Our results are largely consistent with the hypothesis that G proteins passively follow bulk endocytic flow of membrane and suggest that at least some G proteins are recycled to the plasma membrane. Our imaging results also show that G proteins are apparently more abundant on late endosomes and lysosomes than on early endosomes, suggesting that they become concentrated as late endosomes mature. We cannot exclude the possibility that heterotrimers traffic between membrane compartments by mechanisms other than vesicular trafficking (Saini, Chisari, & Gautam, 2009). However, even if this is the case our conclusions that G proteins are internalized inefficiently and are present at lower density on most intracellular membranes are still valid. The cell lines we developed should prove useful for answering additional questions related to G protein regulation, such as possible non-vesicular translocation due to palmitate turnover (Saini, Chisari, & Gautam, 2009; Wedegaertner & Bourne, 1994), the role of ubiquitination (Dohlman & Campbell, 2019), and localization in subcompartments not studied here.

Our study is not without limitations. Our labeling strategy was designed to interfere as little as possible with heterotrimer function, but we cannot rule out the possibility that the tags we used to visualize and track G proteins had some influence on their trafficking. By labeling Gβ_1_ subunits we cannot directly distinguish heterotrimers from free Gβγ dimers, complicating interpretation. This strategy also does not allow us to resolve heterotrimers containing different Gα subunits. It is quite possible that heterotrimers containing different Gα subunits could be subject to different trafficking mechanisms. Our conclusion that GPCR activation has no lasting effect on the subcellular distribution of G proteins rests on three representative receptors, chosen because they activate three of the four major G protein families. It is possible that other receptors will influence G protein distribution using mechanisms not shared by the receptors we studied. Finally, our study was limited to a single non-differentiated cell type. It would not be surprising to find that differentiated cells have mechanisms to regulate G protein trafficking and distribution that are not shared by the model cells we used (Calebiro et al., 2009; Kotowski et al., 2011; Lin et al., 2024; Puri et al., 2022).

In summary, here we show that heterotrimeric G proteins are more abundant on the plasma membrane than on any intracellular compartment where they are thought to be important for signaling. Our results are likely to have functional implications for signaling from intracellular compartments. Receptor-G protein coupling is thought to be rate-limited by collision and G protein abundance (Hein et al., 2005), and decreasing G protein expression is known to impair downstream signaling (Gabay et al., 2011; Schwindinger et al., 1997). Signaling from endosomes and other compartments may thus be disadvantaged by a low density of G proteins. Further studies are warranted to examine the stoichiometry of receptors, G proteins, regulators, and effectors in different subcellular compartments and how this affects signaling.

## Materials and Methods

### Cell culture and transfection.

Human embryonic kidney HEK 293 cells (ATCC; CRL-1573) were propagated in 100 mm dishes, on 6-well plates, or on 25 mm round coverslips in high glucose DMEM (Cytiva) and 10% fetal bovine serum (Cytiva) supplemented with penicillin streptomycin (Gibco). HEK 293T cells stably expressing mNG2(1-10) (Cho et al., 2022) were kindly supplied by Manuel Leonetti (Chan Zuckerberg Biohub San Francisco). Cells were transfected in growth medium using linear polyethyleneimine MAX (Polysciences) at a nitrogen/phosphate ratio of 20 and were used for experiments 24-48 hours later. Up to 3.0 μg of plasmid DNA was transfected in each well of a 6-well plate.

### Gene editing.

Ribonucleoprotein (RNP) complexes were assembled *in vitro* in IDT nuclease free duplex buffer from Alt-R^™^ crRNA, Alt-R^™^ tracrRNA and Alt-R^™^ S.p. Cas9 Nuclease V3 purchased from Integrated DNA Technologies (IDT). RNP and repair ssODNs (dissolved in nuclease-free water) were added to single-cell suspensions (10 μl of 1.2×10^4^ cells μl^−1^) and electroporated using a Neon^™^ Transfection device (Invitrogen) following the manufacturer’s instructions. Cells were expanded and diluted into 48-well plates and grown for 3 weeks. Wells containing single cell colonies were duplicated into 12-well plates and screened for HiBit expression by mixing crude lysates with purified LgBit protein (Promega) and measuring luminescence in the presence of 5 μM furimazine. After clone expansion genomic DNA was extracted using the GeneJET Genomic DNA purification kit (ThermoFisher) and used as a template for amplicon sequencing. Sequencing primers were designed to span the editing site and to produce amplicons less than 500 base pairs in length. Amplicon sequencing was performed by Azenta Life Sciences (Amplicon-EZ) and analyzed using CRISPResso2. Cell lines used for experiments had correctly edited alleles but were hemizygous due to competing repair mechanisms. The human *GNB1* gene was targeted at a site corresponding to the N-terminus of the Gβ_1_ protein; the sequence 5’-TGAGTGAGCTTGACCAGTTA-3’ was incorporated into the crRNA. The ssODN homology-directed repair (HDR) template sequence for mNG-β_1_ cells was: ATCTCACATTCTTGAAGGTGGCATTGAAGAGCACTAAGATCGGAAGATG**ACCGAGCTCAACTTCAAGGAGTGGCAAAAGGCCTTTACCGATATGATG***GGCGGAAGCGGT***GTGTCCGGCTGGCGGCTGTTCAAGAAGATTTCT***GGCGGAAG*CAGTGAGCTTGACCAGCTTAGACAGGAGGCCGAGCAACTTAAGAACCAGA, with the mNG2(11) and HiBit tag sequences in **bold** font, and GGSG and GGS linkers in *italic* font. The repair template sequence for HiBit-α_1_ cells was: TTTCAGATCTCACATTCTTGAAGGTGGCATTGAAGAGCACTAAGATCGGAAG**ATGGTGAGCGGCTGGCGGCTGTTCAAGAAGATTAGC***GGCGGAAGCGGTA*GTGAGCTTGACCAGCTTAGACAGGAGGCCGAGCAACTTAAGAACCAGATTCGAG, with the HiBit tag sequence in **bold** font, and GGSG linker in *italic* font. For both ssODNs a silent mutation (underlined sequence) was introduced to ablate the PAM site.

### SDS-PAGE.

Pelleted cells were mixed with Laemmli buffer (Bio-Rad), and proteins were separated on 4 to 15% SDS polyacrylamide gradient gels (Bio-Rad) then transferred to polyvinylidene difluoride (PVDF) membranes (Millipore Sigma). HiBit-tagged proteins were detected using the NanoGlo HiBit Blotting kit (Promega) following the manufacturer’s instructions, and membranes were imaged using an Amersham Imager 600.

### Plasmids.

The following plasmids were used as received from Addgene: mRuby-Golgi-7 (GalT; #55865), mRuby2-Rab5a-7 (#55911), mCherry-Rab7a-7 (#55127), mCherry-Rab11a-7 (#55124), pmCherry-2xFYVE (#140050). Venus-2xFYVE was made by replacing mCherry in pmCherry-2xFYVE with Venus using *Nhel* and *BsrGI*. mRuby2-MOA was made by replacing Venus in Venus-MOA using *NheI* and *BglII*. mRuby2-PTP1b was made by replacing Venus in Venus-PTP1b using *NheI* and *BsrGI*. CMV-LgBit was made by amplifying LgBit from pBiT1.1-N (Promega) and ligating into pcDNA3.1 (+) using *HindIII* and *XhoI*. SNAPf-β_2_AR, SNAPf-D2R, SNAPf-M3R and D2S-Nluc were kindly provided by Jonathan Javitch (Columbia University). Venus-Kras, Venus-PTP1b, Venus-MOA, Venus-rab5a, Venus-rab7a, Venus-rab11a and memGRKct-Venus were described previously (Hollins et al., 2009; Lan et al., 2012). All plasmids were verified by automated sequencing.

### Imaging.

Imaging was performed on a Leica SP8 laser scanning confocal microscope using a 63× 1.40 NA oil immersion objective. Cells grown on 25 mm round coverslips were transferred to a steel imaging chamber and imaged in HEPES Imaging (HI) buffer which contained 150 mM NaCl, 10 mM NaHEPES, 5 mM glucose, 2.5 mM KCl, 1.2 mM CaCl_2_, 1 mM MgCl_2_ (pH 7.2). All imaging was carried out at room temperature with the exception of the experiment shown in *SI Appendix* Figure S9, which was carried out at 37°C. For colocalization of mNG-β_1_ and red organelle markers 0.2 μg of each marker was transfected per coverslip; mNG-α1 was excited at 488 nm and detected at 495-545 nm, and red markers were excited at 552 nm and detected at 565-665 nm. Lysosomes were stained with either LysoView 633 (Biotium; 1:1,000 in growth medium for 15 minutes at 37°C) or 10,000 m.w. CF 640 dextran (Biotium; 25 μg ml^−1^ overnight at 37°C followed by a 60-minute chase); both dyes were excited at 638 nm and detected at 650-700 nm. For simultaneous imaging of mNG-β_1_, FM4-64 and CellMask Deep Red, cells were placed in HI buffer containing 1:1,000 CellMask Deep Red (Invitrogen) for 15 minutes at room temperature, then returned to culture medium containing 5 μM FM4-64 (a.k.a. SynaptoRed; Calbiochem) and incubated at 37°C for 15 minutes. Imaging was then performed in HI buffer containing 5 μM FM4-64. For simultaneous imaging of mNG-β_1_, FM4-64 and β_2_AR, cells were transfected with 1 μg of SNAPf-β_2_AR, stained in HI buffer containing 5 μM SNAP-Surface Alexa 647 (New England Biolabs) for 15 minutes at room temperature, then returned to culture medium containing 10 μM isoproterenol and 5 μM FM4-64 and incubated at 37°C for 15 minutes. Imaging was then performed in HI buffer containing 5 μM FM4-64. CellMask Deep Red and SNAP-Surface Alexa 647 were excited at 638 nm and detected at 650-700 nm; mNG-β_1_ and FM4-64 were excited at 488 nm and detected at 500-570 nm and 675-755 nm, respectively.

### Image analysis.

Signal/background ratios for the plasma membrane and endosomes (Figure 3C) were calculated using mean fluorescence values from rectangular (for the plasma membrane) and round (for endosomes) regions of interest (ROIs) surrounding the structures and nearby cytosol (for background). A single plasma membrane ROI and 1-3 endosome ROIs were sampled per cell/image. Mean fluorescence intensity line profiles were extracted from 3 μm lines centered on vesicles as absolute fluorescence intensity (Figure 3B), or fluorescence intensity normalized to the mean intensity of a nearby section of plasma membrane (Figure 4C and Figure 5C). Vesicles that contained and did not contain internalized receptors were compared (Figure 5D) by dividing the peak mNG-β_1_ signal by the peak FM4-64 signal for each vesicle; both signals were first normalized to their respective plasma membrane signals and subjected to background subtraction. A single vesicle was sampled per cell/image. All image analysis was carried out using ImageJ and raw images. For construction of figures images were exported as .TIF files with or without uniform contrast enhancement applied by ImageJ.

### BRET.

For bystander BRET mapping of HiBit-β_1_ localization cells were transfected in 6-well plates with 1 μg per well of a Venus-tagged compartment marker and 0.1 μg per well of CMV-LgBit. For measurements cells were resuspended in Dulbecco’s phosphate buffered saline (DPBS). For long-term agonist stimulation (Figure 5E) HiBit-β_1_ cells expressing CMV-LgBit (0.1 μg per well) and either SNAPf-β2AR, SNAPf-D2R or SNAPf-M3R (0.5 μg per well) were incubated with agonist for 30 minutes in the incubator, then washed and resuspended in DPBS containing antagonist prior to reading BRET. Agonists were isoproterenol (10 μM), dopamine (100 μM) and acetylcholine (100 μM); antagonists were ICI 118551 (10 μM), haloperidol (10 μM) and atropine (10 μM); all small molecule ligands were obtained from Millipore Sigma or Cayman Chemical. For functional validation of mNG-β_1_ cells, D2R-Nluc (50 ng per well) was transfected, and cells were resuspended in permeabilization buffer (KPS) containing 140 mM KCl, 10 mM NaCl, 1 mM MgCl_2_, 0.1 mM Potassium EGTA, 20 mM NaHEPES (pH 7.2), 10 μ ml^−1^ high-purity digitonin and 2U ml^−1^ apyrase. Kinetic BRET measurements were made from permeabilized cells during sequential injection of dopamine (100 μM) and GDP (100 μM). For functional validation of HiBit-β_1_ cells, SNAPf-β2AR, SNAPf-D2R or SNAPf-M3R (0.5 μg per well), CMV-LgBit (0.1 μg per well) and memGRK3ct-Venus (0.5 μg per well) were transfected, and cells were resuspended in DPBS. Kinetic BRET measurements were made from intact cells during sequential injection of agonists and antagonists at the concentrations listed above. All BRET measurements were made in buffer solutions containing the substrate furimazine (Promega or ChemShuttle; 1:1,000 from a 5 mM stock dissolved in 90% ethanol/10% glycerol). Steady-state BRET and luminescence measurements were made using a Mithras LB940 photon-counting plate reader (Berthold Technologies GmbH) running MicroWin2000 software. Kinetic BRET measurements were made using a Polarstar Optima plate reader (BMG Labtech) running BMG Optima version 2.20R2 software. Raw BRET signals were calculated as the emission intensity at 520–545 nm divided by the emission intensity at 475–495 nm. Net BRET signals were calculated as the raw BRET signal minus the raw BRET signal measured from cells expressing only the donor.

## Statistical analysis.

All statistical testing was carried out using GraphPad Prism version 10.1.1. Comparison of mNG signals in vesicles with and without receptors (Figure 5D) was made using an unpaired t-test. Comparison of endosome bystander signals with and without agonist treatment (Figure 5E) was made using paired t-tests with a false discovery rate (FDR) of 1% (method of Benjamini, Krieger and Yekutieli).

## Data availability.

All study data are included in the article and source data files. Cell lines and plasmids generated for this study are freely available upon request from the corresponding author. No unique code or software was used for the study.

## Supplementary Material

1

## References

[R1] Akgoz M., Kalyanaraman V., & Gautam N. (2004). Receptor-mediated reversible translocation of the G protein betagamma complex from the plasma membrane to the Golgi complex. J Biol Chem, 279(49), 51541–51544. 10.1074/jbc.M410639200 15448129

[R2] Benovic J. L., Bouvier M., Caron M. G., & Lefkowitz R. J. (1988). Regulation of adenylyl cyclase-coupled beta-adrenergic receptors. Annu Rev Cell Biol, 4, 405–428. 10.1146/annurev.cb.04.110188.002201 2848553

[R3] Betz W. J., Mao F., & Smith C. B. (1996). Imaging exocytosis and endocytosis. Curr Opin Neurobiol, 6(3), 365–371. 10.1016/s0959-4388(96)80121-8 8794083

[R4] Bowman S. L., Shiwarski D. J., & Puthenveedu M. A. (2016). Distinct G protein-coupled receptor recycling pathways allow spatial control of downstream G protein signaling. J Cell Biol, 214(7), 797–806. 10.1083/jcb.201512068 27646272 PMC5037407

[R5] Calebiro D., Nikolaev V. O., Gagliani M. C., de Filippis T., Dees C., Tacchetti C., Persani L., & Lohse M. J. (2009). Persistent cAMP-signals triggered by internalized G-protein-coupled receptors. PLoS biology, 7(8), e1000172–e1000172. 10.1371/journal.pbio.1000172 19688034 PMC2718703

[R6] Calebiro D., Nikolaev V. O., Persani L., & Lohse M. J. (2010). Signaling by internalized G-protein-coupled receptors. Trends Pharmacol Sci, 31(5), 221–228. 10.1016/j.tips.2010.02.002 20303186

[R7] Chisari M., Saini D. K., Kalyanaraman V., & Gautam N. (2007). Shuttling of G protein subunits between the plasma membrane and intracellular membranes. The Journal of biological chemistry, 282(33), 24092–24098. 10.1074/jbc.M704246200 17576765 PMC2238717

[R8] Cho N. H., Cheveralls K. C., Brunner A. D., Kim K., Michaelis A. C., Raghavan P., Kobayashi H., Savy L., Li J. Y., Canaj H., Kim J. Y. S., Stewart E. M., Gnann C., McCarthy F., Cabrera J. P., Brunetti R. M., Chhun B. B., Dingle G., Hein M. Y., … Leonetti M. D. (2022). OpenCell: Endogenous tagging for the cartography of human cellular organization. Science, 375(6585), eabi6983. 10.1126/science.abi6983 35271311 PMC9119736

[R9] Crouthamel M., Thiyagarajan M. M., Evanko D. S., & Wedegaertner P. B. (2008). N-terminal polybasic motifs are required for plasma membrane localization of Galpha(s) and Galpha(q). Cellular signalling, 20(10), 1900–1910. 10.1016/j.cellsig.2008.06.019 18647648 PMC2603300

[R10] DeGroot A. C. M., Busch D. J., Hayden C. C., Mihelic S. A., Alpar A. T., Behar M., & Stachowiak J. C. (2018). Entropic Control of Receptor Recycling Using Engineered Ligands. Biophys J, 114(6), 1377–1388. 10.1016/j.bpj.2018.01.036 29590595 PMC5883623

[R11] Doherty G. J., & McMahon H. T. (2009). Mechanisms of endocytosis. Annu Rev Biochem, 78, 857–902. 10.1146/annurev.biochem.78.081307.110540 19317650

[R12] Dohlman H. G., & Campbell S. L. (2019). Regulation of large and small G proteins by ubiquitination. J Biol Chem, 294(49), 18613–18623. 10.1074/jbc.REV119.011068 31645437 PMC6901297

[R13] Eichel K., & von Zastrow M. (2018). Subcellular Organization of GPCR Signaling. Trends Pharmacol Sci, 39(2), 200–208. 10.1016/j.tips.2017.11.009 29478570 PMC5830169

[R14] Evanko D. S., Thiyagarajan M. M., & Wedegaertner P. B. (2000). Interaction with Gbetagamma is required for membrane targeting and palmitoylation of Galpha(s) and Galpha(q). The Journal of biological chemistry, 275(2), 1327–1336. http://www.ncbi.nlm.nih.gov/pubmed/10625681 10625681 10.1074/jbc.275.2.1327

[R15] Feng S., Sekine S., Pessino V., Li H., Leonetti M. D., & Huang B. (2017). Improved split fluorescent proteins for endogenous protein labeling. Nat Commun, 8(1), 370. 10.1038/s41467-017-00494-8 28851864 PMC5575300

[R16] Ferrandon S., Feinstein T. N., Castro M., Wang B., Bouley R., Potts J. T., Gardella T. J., & Vilardaga J. P. (2009). Sustained cyclic AMP production by parathyroid hormone receptor endocytosis. Nat Chem Biol, 5(10), 734–742. 10.1038/nchembio.206 19701185 PMC3032084

[R17] Gabay M., Pinter M. E., Wright F. A., Chan P., Murphy A. J., Valenzuela D. M., Yancopoulos G. D., & Tall G. G. (2011). Ric-8 proteins are molecular chaperones that direct nascent G protein α subunit membrane association. Sci Signal, 4(200), ra79. 10.1126/scisignal.2002223 22114146 PMC3870195

[R18] Gilman A. G. (1987). G proteins: transducers of receptor-generated signals. Annual review of biochemistry, 56, 615–649. 10.1146/annurev.bi.56.070187.003151 3113327

[R19] Hammond G. R., & Balla T. (2015). Polyphosphoinositide binding domains: Key to inositol lipid biology. Biochim Biophys Acta, 1851(6), 746–758. 10.1016/j.bbalip.2015.02.013 25732852 PMC4380703

[R20] Hanyaloglu A. C., & von Zastrow M. (2008). Regulation of GPCRs by endocytic membrane trafficking and its potential implications. Annu Rev Pharmacol Toxicol, 48, 537–568. 18184106 10.1146/annurev.pharmtox.48.113006.094830

[R21] Hein P., Frank M., Hoffmann C., Lohse M. J., & Bunemann M. (2005). Dynamics of receptor/G protein coupling in living cells. Embo J, 24(23), 4106–4114. http://www.ncbi.nlm.nih.gov/entrez/query.fcgi?cmd=Retrieve&db=PubMed&dopt=Citation&list_uids=16292347 16292347 10.1038/sj.emboj.7600870 PMC1356310

[R22] Hewavitharana T., & Wedegaertner P. B. (2012). Non-canonical signaling and localizations of heterotrimeric G proteins. Cellular signalling, 24(1), 25–34. 10.1016/j.cellsig.2011.08.014 21907280 PMC3205251

[R23] Hillenbrand M., Schori C., Schöppe J., & Plückthun A. (2015). Comprehensive analysis of heterotrimeric G-protein complex diversity and their interactions with GPCRs in solution. Proc Natl Acad Sci U S A, 112(11), E1181–1190. 10.1073/pnas.1417573112 25733868 PMC4371982

[R24] Hollins B., Kuravi S., Digby G. J., & Lambert N. A. (2009). The c-terminus of GRK3 indicates rapid dissociation of G protein heterotrimers. Cellular signalling, 21(6). 10.1016/j.cellsig.2009.02.017 PMC2668204 19258039

[R25] Hynes T. R., Mervine S. M., Yost E. A., Sabo J. L., & Berlot C. H. (2004). Live cell imaging of Gs and the beta2-adrenergic receptor demonstrates that both alphas and beta1gamma7 internalize upon stimulation and exhibit similar trafficking patterns that differ from that of the beta2-adrenergic receptor. The Journal of biological chemistry, 279(42), 44101–44112. 10.1074/jbc.M405151200 15297467

[R26] Irannejad R., Pessino V., Mika D., Huang B., Wedegaertner P. B., Conti M., & von Zastrow M. (2017). Functional selectivity of GPCR-directed drug action through location bias. Nature chemical biology, 13(7), 799–806. 10.1038/nchembio.2389 28553949 PMC5733145

[R27] Irannejad R., Tomshine J. C., Tomshine J. R., Chevalier M., Mahoney J. P., Steyaert J., Rasmussen S. G., Sunahara R. K., El-Samad H., Huang B., & von Zastrow M. (2013). Conformational biosensors reveal GPCR signalling from endosomes. Nature, 495(7442), 534–538. 10.1038/nature12000 23515162 PMC3835555

[R28] Kotowski S. J., Hopf F. W., Seif T., Bonci A., & von Zastrow M. (2011). Endocytosis promotes rapid dopaminergic signaling. Neuron, 71(2), 278–290. 10.1016/j.neuron.2011.05.036 21791287 PMC3417347

[R29] Krumins A. M., & Gilman A. G. (2006). Targeted knockdown of G protein subunits selectively prevents receptor-mediated modulation of effectors and reveals complex changes in non-targeted signaling proteins. J Biol Chem, 281(15), 10250–10262. 10.1074/jbc.M511551200 16446365

[R30] Lan T.-H., Liu Q., Li C., Wu G., & Lambert N. A. (2012). Sensitive and high resolution localization and tracking of membrane proteins in live cells with BRET. Traffic (Copenhagen, Denmark), 13(11), 1450–1456. 10.1111/j.1600-0854.2012.01401.x 22816793 PMC3889717

[R31] Lazar A. M., Irannejad R., Baldwin T. A., Sundaram A. B., Gutkind J. S., Inoue A., Dessauer C. W., & Von Zastrow M. (2020). G protein-regulated endocytic trafficking of adenylyl cyclase type 9. eLife, 9. 10.7554/eLife.58039 PMC7332294 32515353

[R32] Lin T. Y., Mai Q. N., Zhang H., Wilson E., Chien H. C., Yee S. W., Giacomini K. M., Olgin J. E., & Irannejad R. (2024). Cardiac contraction and relaxation are regulated by distinct subcellular cAMP pools. Nat Chem Biol, 20(1), 62–73. 10.1038/s41589-023-01381-8 37474759 PMC10746541

[R33] Marrari Y., Crouthamel M., Irannejad R., & Wedegaertner P. B. (2007). Assembly and Trafficking of Heterotrimeric G Proteins †. Biochemistry, 46(26), 7665–7677. 10.1021/bi700338m 17559193 PMC2527407

[R34] Mullershausen F., Zecri F., Cetin C., Billich A., Guerini D., & Seuwen K. (2009). Persistent signaling induced by FTY720-phosphate is mediated by internalized S1P1 receptors. Nat Chem Biol, 5(6), 428–434. 10.1038/nchembio.173 19430484

[R35] Nakano A. (2022). The Golgi Apparatus and its Next-Door Neighbors. Front Cell Dev Biol, 10, 884360. 10.3389/fcell.2022.884360 35573670 PMC9096111

[R36] Pierce K. L., Premont R. T., & Lefkowitz R. J. (2002). Seven-transmembrane receptors. Nat Rev Mol Cell Biol, 3(9), 639–650. 12209124 10.1038/nrm908

[R37] Pinilla-Macua I., Watkins S. C., & Sorkin A. (2016). Endocytosis separates EGF receptors from endogenous fluorescently labeled HRas and diminishes receptor signaling to MAP kinases in endosomes. Proc Natl Acad Sci U S A, 113(8), 2122–2127. 10.1073/pnas.1520301113 26858456 PMC4776482

[R38] Puri N. M., Romano G. R., Lin T. Y., Mai Q. N., & Irannejad R. (2022). The organic cation transporter 2 regulates dopamine D1 receptor signaling at the Golgi apparatus. eLife, 11. 10.7554/eLife.75468 PMC9098220 35467530

[R39] Ransnäs L. A., Svoboda P., Jasper J. R., & Insel P. A. (1989). Stimulation of beta-adrenergic receptors of S49 lymphoma cells redistributes the alpha subunit of the stimulatory G protein between cytosol and membranes. Proceedings of the National Academy of Sciences of the United States of America, 86(20), 7900–7903. http://www.pubmedcentral.nih.gov/articlerender.fcgi?artid=298179&tool=pmcentrez&rendertype=abstract 2554294 10.1073/pnas.86.20.7900 PMC298179

[R40] Saini D. K., Chisari M., & Gautam N. (2009). Shuttling and translocation of heterotrimeric G proteins and Ras. Trends in pharmacological sciences, 30(6), 278–286. 10.1016/j.tips.2009.04.001 19427041 PMC3097116

[R41] Saini D. K., Kalyanaraman V., Chisari M., & Gautam N. (2007). A family of G protein βγ subunits translocate reversibly from the plasma membrane to endomembranes on receptor activation. J Biol Chem, 282(33), 24099–24108. 10.1074/jbc.M701191200 17581822 PMC2238721

[R42] Scarselli M., & Donaldson J. G. (2009). Constitutive internalization of G protein-coupled receptors and G proteins via clathrin-independent endocytosis. The Journal of biological chemistry, 284(6), 3577–3585. 10.1074/jbc.M806819200 19033440 PMC2635037

[R43] Schwindinger W. F., Reese K. J., Lawler A. M., Gearhart J. D., & Levine M. A. (1997). Targeted disruption of Gnas in embryonic stem cells. Endocrinology, 138(10), 4058–4063. 10.1210/endo.138.10.5439 9322912

[R44] Schwinn M. K., Machleidt T., Zimmerman K., Eggers C. T., Dixon A. S., Hurst R., Hall M. P., Encell L. P., Binkowski B. F., & Wood K. V. (2018). CRISPR-Mediated Tagging of Endogenous Proteins with a Luminescent Peptide. ACS Chemical Biology, 13(2), 467–474. 10.1021/acschembio.7b00549 28892606

[R45] Shahinian S., & Silvius J. R. (1995). Doubly-lipid-modified protein sequence motifs exhibit long-lived anchorage to lipid bilayer membranes. Biochemistry, 34(11), 3813–3822. 10.1021/bi00011a039 7893678

[R46] Slepak V. Z., & Hurley J. B. (2008). Mechanism of light-induced translocation of arrestin and transducin in photoreceptors: interaction-restricted diffusion. IUBMB life, 60(1), 2–9. 10.1002/iub.7 18379987 PMC2717607

[R47] Stenmark H. (2009). Rab GTPases as coordinators of vesicle traffic. Nat Rev Mol Cell Biol, 10(8), 513–525. 10.1038/nrm2728 19603039

[R48] Surve S., Watkins S. C., & Sorkin A. (2021). EGFR-RAS-MAPK signaling is confined to the plasma membrane and associated endorecycling protrusions. J Cell Biol, 220(11). 10.1083/jcb.202107103 PMC8563293 34515735

[R49] Takida S., & Wedegaertner P. B. (2003). Heterotrimer Formation, Together with Isoprenylation, Is Required for Plasma Membrane Targeting of Gbeta gamma. Journal of Biological Chemistry, 278(19), 17284–17290. 10.1074/jbc.M213239200 12609996

[R50] Tsutsumi R., Fukata Y., Noritake J., Iwanaga T., Perez F., & Fukata M. (2009). Identification of G protein alpha subunit-palmitoylating enzyme. Mol Cell Biol, 29(2), 435–447. 10.1128/mcb.01144-08 19001095 PMC2612512

[R51] Tsvetanova N. G., Irannejad R., & von Zastrow M. (2015). GPCR signaling via heterotrimeric G proteins from endosomes. Journal of Biological Chemistry, 290(11), jbc.R114.617951-jbc.R617114.617951. 10.1074/jbc.R114.617951 PMC4358092 25605726

[R52] von Zastrow M., & Kobilka B. K. (1992). Ligand-regulated internalization and recycling of human beta 2-adrenergic receptors between the plasma membrane and endosomes containing transferrin receptors. J Biol Chem, 267(5), 3530–3538. 1371121

[R53] Wedegaertner P. B. (2012). G protein trafficking. Subcellular Biochemistry, 63, 193–223. 10.1007/978-94-007-4765-411 23161140 PMC5936075

[R54] Wedegaertner P. B., & Bourne H. R. (1994). Activation and depalmitoylation of Gs alpha. Cell, 77(7), 1063–1070. http://www.ncbi.nlm.nih.gov/pubmed/7912657 7912657 10.1016/0092-8674(94)90445-6

[R55] Wedegaertner P. B., Bourne H. R., & von Zastrow M. (1996). Activation-induced subcellular redistribution of Gs alpha. Molecular biology of the cell, 7(8), 1225–1233. http://www.pubmedcentral.nih.gov/articlerender.fcgi?artid=275974&tool=pmcentrez&rendertype=abstract 8856666 10.1091/mbc.7.8.1225 PMC275974

