# Supplementary material for: Visualization of endogenous G proteins on endosomes and other organelles
Source: bioRxiv. 2024 Mar 7:2024.03.05.583500. Preprint. [Version 2] doi: 10.1101/2024.03.05.583500 (PMC10942389; doi:10.1101/2024.03.05.583500)
Supplement: Supplementary file 1 [file NIHPP2024.03.05.583500v2-supplement-1.pdf]

bioRxiv preprint doi: https://doi.org/10.1101/2024.03.05.583500; this version posted March 7, 2024. The copyright holder for this preprint (which was not certified by peer review) is the author/funder, who has granted bioRxiv a license to display the preprint in perpetuity. It is made available under aCC-BY 4.0 International license.

Figure 2 - figure supplement 1

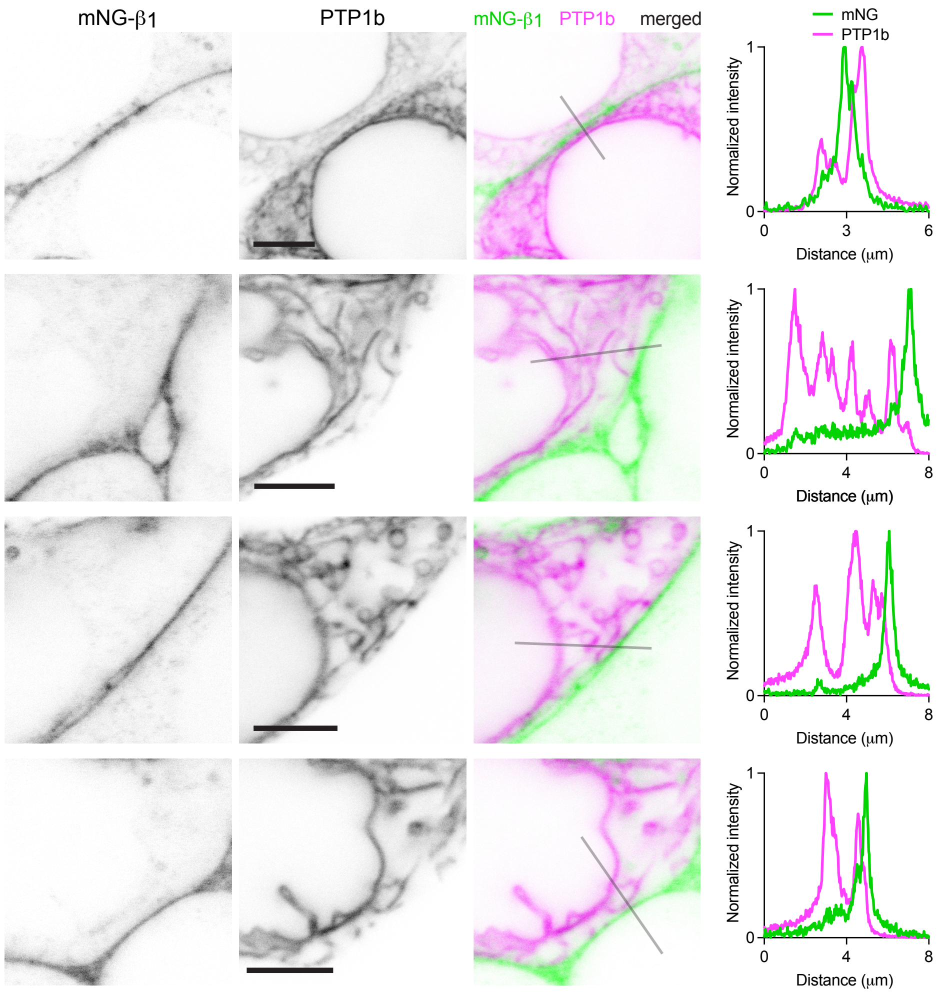

G proteins are not abundant on the endoplasmic reticulum (ER). Exemplary images of mNG-β_1 cells coexpressing the ER marker mRuby2-PTP1b. Intensity line profiles depict fluorescence intensity normalized to the maximum value in each channel; scale bars are 5 μm.

bioRxiv preprint doi: https://doi.org/10.1101/2024.03.05.583500; this version posted March 7, 2024. The copyright holder for this preprint (which was not certified by peer review) is the author/funder, who has granted bioRxiv a license to display the preprint in perpetuity. It is made available under aCC-BY 4.0 International license.

Figure 2 - figure supplement 2

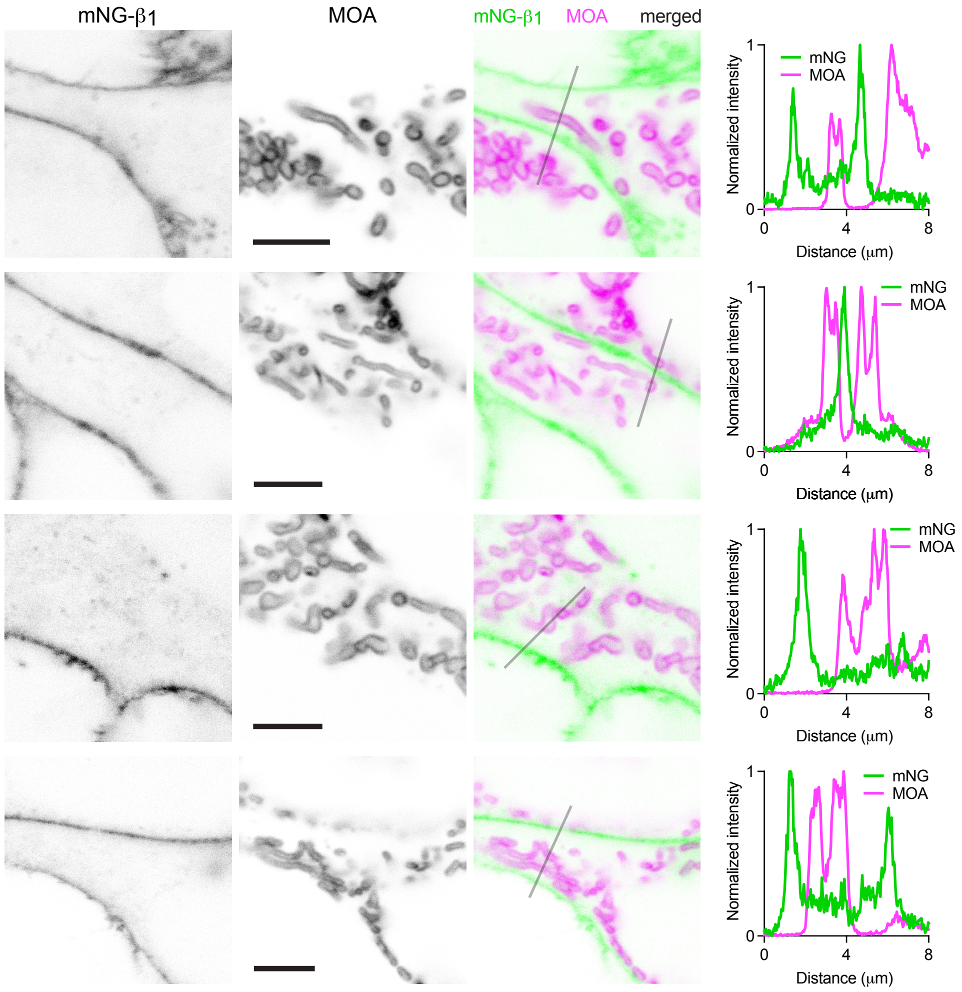

G proteins are not abundant on mitochondria (MT). Exemplary images of mNG-β_1 cells coexpressing the MT marker mRuby2-MOA. Intensity line profiles depict fluorescence intensity normalized to the maximum value in each channel; scale bars are 5 μm.

bioRxiv preprint doi: https://doi.org/10.1101/2024.03.05.583500; this version posted March 7, 2024. The copyright holder for this preprint (which was not certified by peer review) is the author/funder, who has granted bioRxiv a license to display the preprint in perpetuity. It is made available under aCC-BY 4.0 International license.

Figure 2 - figure supplement 3

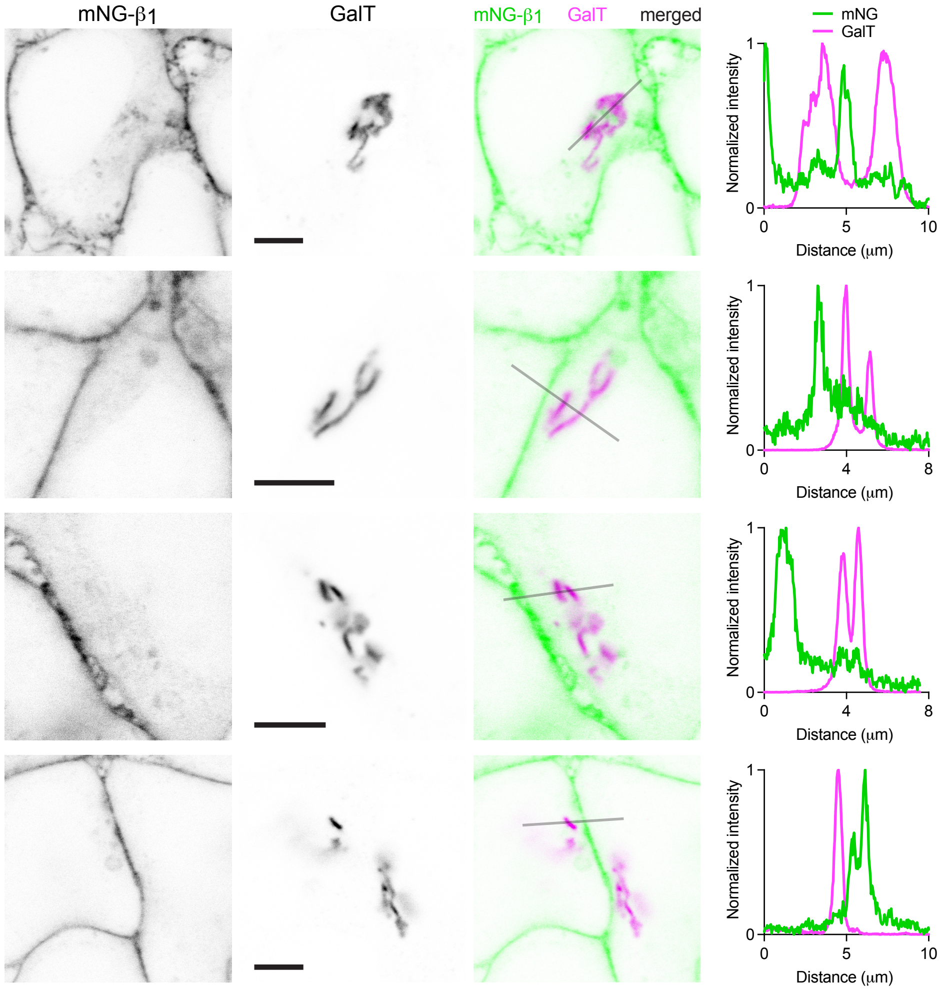

G proteins are not abundant on the medial Golgi apparatus (GA). Exemplary images of mNG-β_1 cells coexpressing the GA marker mRuby2-Golgi-7 (GalT). Intensity line profiles depict fluorescence intensity normalized to the maximum value in each channel; scale bars are 5 μm.

bioRxiv preprint doi: https://doi.org/10.1101/2024.03.05.583500; this version posted March 7, 2024. The copyright holder for this preprint (which was not certified by peer review) is the author/funder, who has granted bioRxiv a license to display the preprint in perpetuity. It is made available under aCC-BY 4.0 International license.

Figure 3 - figure supplement 1

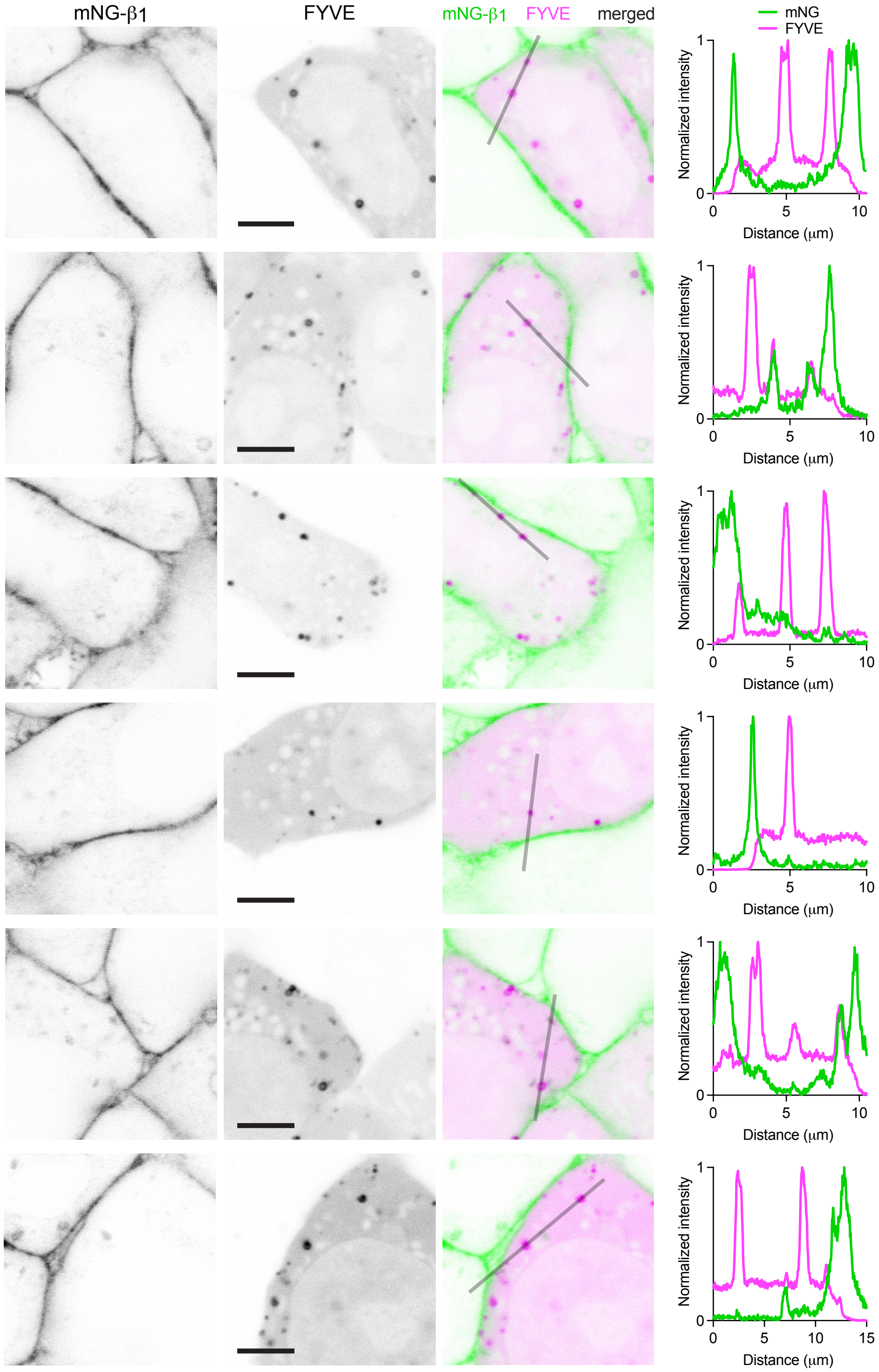

G proteins colocalize with the early endosome marker FYVE on some endosomes. Exemplary images of mNG-β_1 cells coexpressing the early endosome marker pmCherry-2xFYVE (FYVE). Intensity line profiles depict fluorescence intensity normalized to the maximum value in each channel; scale bars are 5 μm.

bioRxiv preprint doi: https://doi.org/10.1101/2024.03.05.583500; this version posted March 7, 2024. The copyright holder for this preprint (which was not certified by peer review) is the author/funder, who has granted bioRxiv a license to display the preprint in perpetuity. It is made available under aCC-BY 4.0 International license.

Figure 3 - figure supplement 2

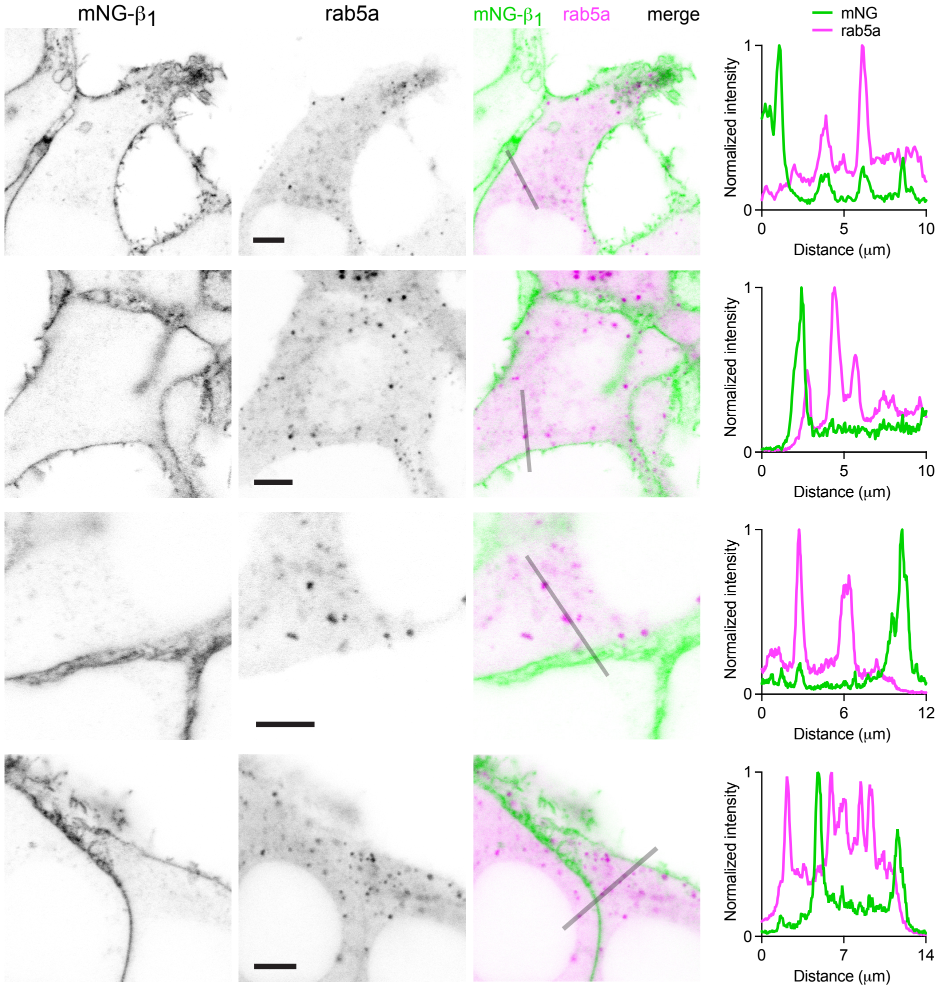

G proteins colocalize with the early endosome marker rab5a on some endosomes. Exemplary images of mNG-β_1 cells coexpressing the early endosome marker mRuby2-rab5a (rab5a). Intensity line profiles depict fluorescence intensity normalized to the maximum value in each channel; scale bars are 5 μm.

bioRxiv preprint doi: https://doi.org/10.1101/2024.03.05.583500; this version posted March 7, 2024. The copyright holder for this preprint (which was not certified by peer review) is the author/funder, who has granted bioRxiv a license to display the preprint in perpetuity. It is made available under aCC-BY 4.0 International license.

Figure 3 - figure supplement 3

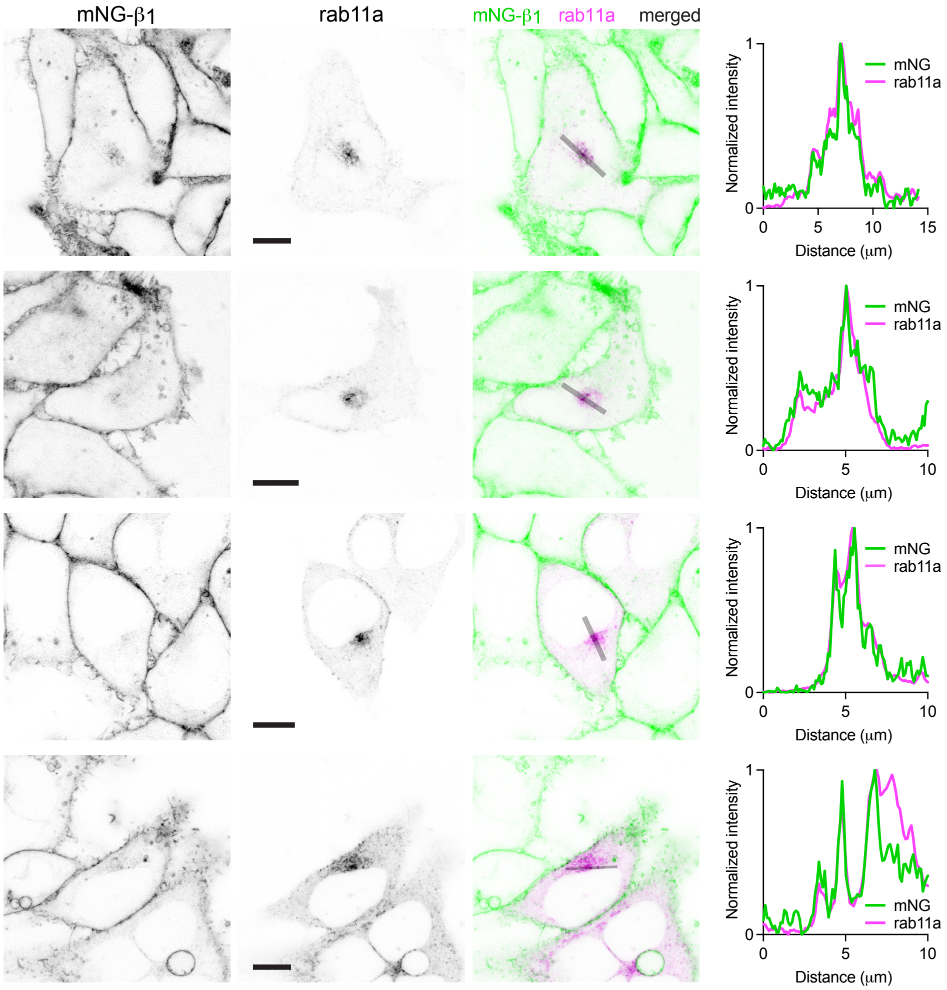

G proteins colocalize with the recycling endosome marker rab11a in a perinuclear region. Exemplary images of mNG-β_1 cells coexpressing the recycling endosome marker mCherry-rab11a (rab11a). Intensity line profiles depict fluorescence intensity normalized to the maximum value in each channel; scale bars are 10 μm.

bioRxiv preprint doi: https://doi.org/10.1101/2024.03.05.583500; this version posted March 7, 2024. The copyright holder for this preprint (which was not certified by peer review) is the author/funder, who has granted bioRxiv a license to display the preprint in perpetuity. It is made available under aCC-BY 4.0 International license.

Figure 3 - figure supplement 4

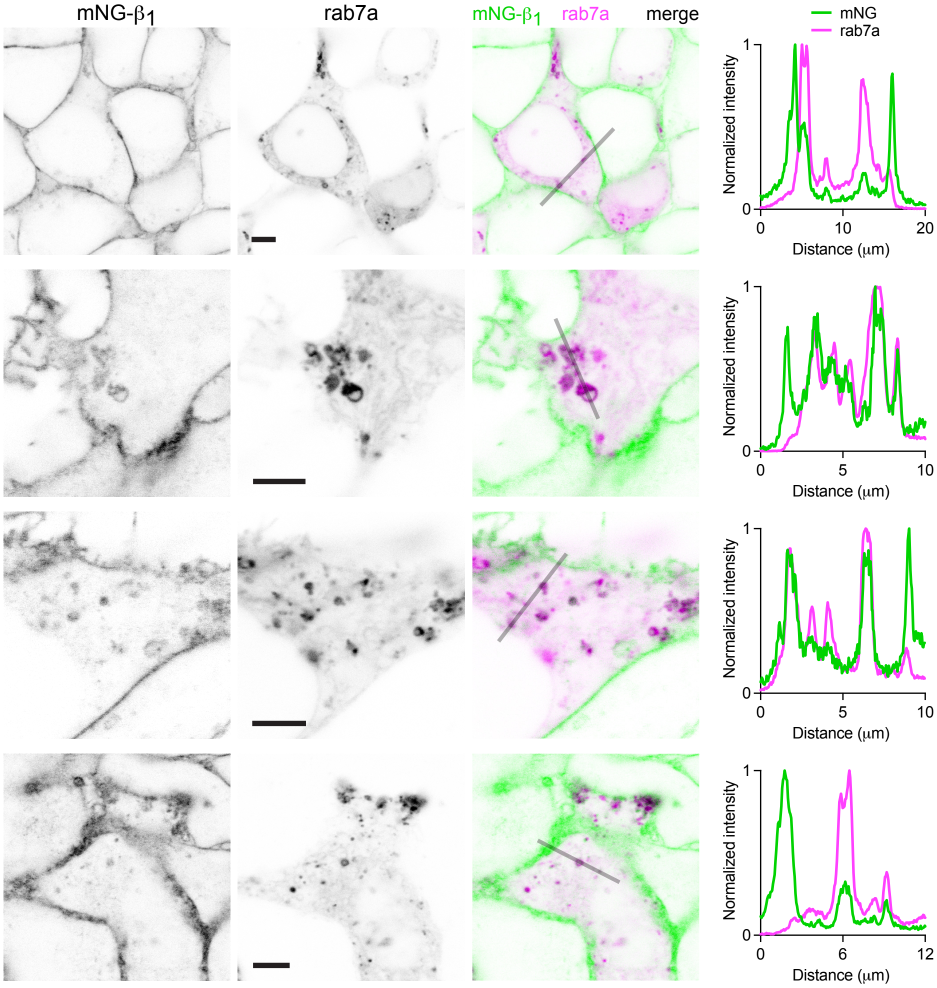

G proteins colocalize with the late endosome marker rab7a on many endosomes. Exemplary images of mNG-β_1 cells coexpressing the late endosome marker mCherry-rab7a (rab7a). Intensity line profiles depict fluorescence intensity normalized to the maximum value in each channel; scale bars are 5 μm.

bioRxiv preprint doi: https://doi.org/10.1101/2024.03.05.583500; this version posted March 7, 2024. The copyright holder for this preprint (which was not certified by peer review) is the author/funder, who has granted bioRxiv a license to display the preprint in perpetuity. It is made available under aCC-BY 4.0 International license.

Figure 3 - figure supplement 5

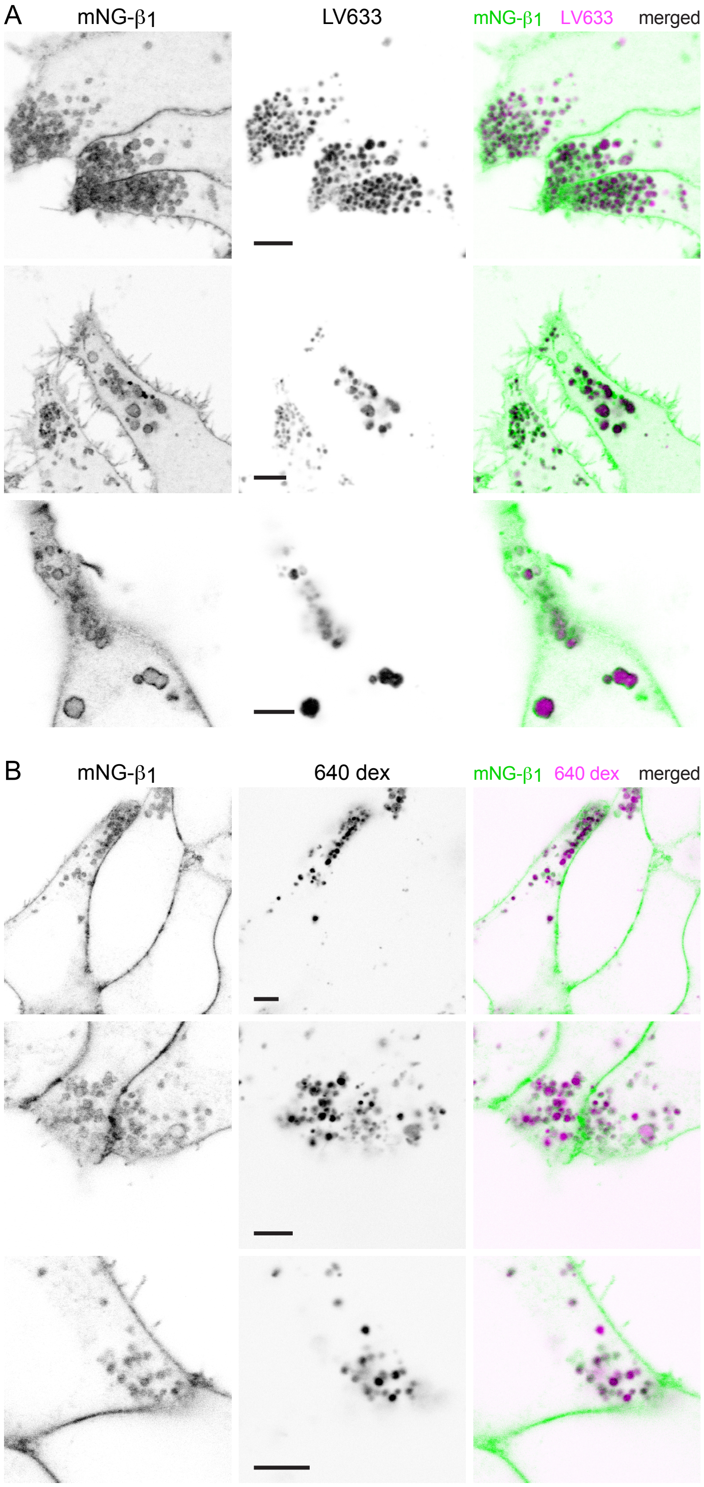

G proteins are abundant on lysosomes. (**A**) Exemplary images of mNG-β_1 cells stained with LysoView 633 (LV633); scale bars are 5 μm. (**B**) Exemplary images of mNG-β_1 cells incubated overnight with 10,000 m.w. CF 640 dextran (640 dex); scale bars are 5 μm.

bioRxiv preprint doi: https://doi.org/10.1101/2024.03.05.583500; this version posted March 7, 2024. The copyright holder for this preprint (which was not certified by peer review) is the author/funder, who has granted bioRxiv a license to display the preprint in perpetuity. It is made available under aCC-BY 4.0 International license.

Figure 4 - figure supplement 1

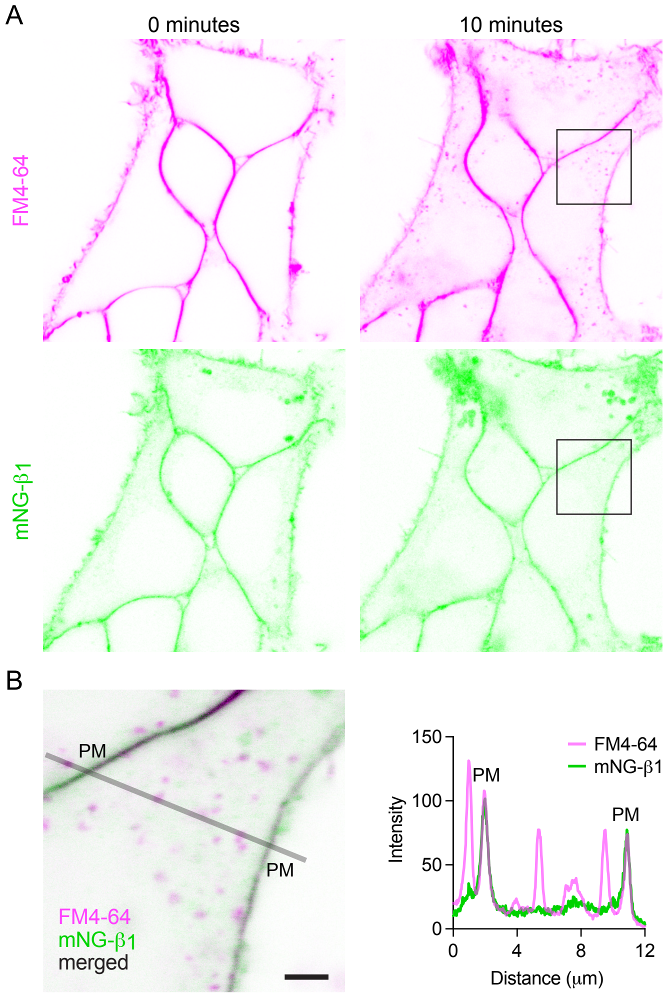

Constitutive endocytosis of G proteins is inefficient. (**A**) Images of mNG-β_1 cells immediately after and 10 minutes after staining with FM4-64. (**B**) An absolute fluorescence intensity line profile for mNG-β_1 and FM4-64; scale bar is 2 μm. In this example absolute fluorescence intensity at the plasma membrane (PM) was similar for the two labels.
